# Effect of huankuile on colon injury in rats with ulcerative colitis by reducing TNF-α and MMP9

**DOI:** 10.1186/s40001-024-01695-w

**Published:** 2024-02-06

**Authors:** Xilinguli Wushouer, Kasimujiang Aximujiang, Nafeisha Kadeer, Abulaiti Aihemaiti, Li Zhong, Kurexi Yunusi

**Affiliations:** 1 Department of Biology, School of Basic Medical Sciences, Xinjiang Medical University, Urumqi, 830017 China; 2 Xinjiang key laboratory of Molecular Biology for endemic diseases, Urumqi , 830054 China; 3 Department of Biochemistry and Molecular Biology, School of Basic Medical Sciences, Xinjiang Medical University, Urumqi, 830017 China; 4 The Functional Center, School of Basic Medical Sciences, Xinjiang Medical University, Urumqi, 830017 China; 5 UygurMedical College, Xinjiang Medical University, Urumqi, 830017 China

**Keywords:** Huankuile, Ulcerative colitis, Inflammatory factor, TNF-α, MMP9

## Abstract

**Objective:**

To explore the mechanism of huankuile (HKL) in colon injury repair in rats with ulcerative colitis (UC).

**Methods:**

Fifty SPF Wistar male rats were divided randomly into a normal group, a negative control group, an HKL intervention group (‘HKL group’) and a 5-aminosalicylic acid intervention group (‘5-ASA group’). After 14 days of intervention with corresponding drugs, pathological scores were obtained using the results of immunohistochemical staining; morphological changes were observed by hematoxylin–eosin staining, and the mRNA expression levels of tumour necrosis factor-α (TNF-α), matrix metalloproteinase 9 (MMP9) and interleukin-13 (IL-13) were detected by real-time quantitative PCR.

**Results:**

After the successful construction of the rat model, it was compared with the rats in the normal group. In the negative group, it was found that the expression of TNF-α and MMP9 was significantly increased in the colonic mucosal epithelia of the rats, the pathological score was significantly increased (*P* < 0.05), and the mRNA expression levels of TNF-α, MMP9 and IL-13 were increased (*P* < 0.05). After treatment with HKL, the colonic morphology of the rats returned to normal, the expression of TNF-α and MMP9 in the colonic mucosal epithelium of the rats returned to normal, the pathological score grade was significantly reduced (*P* < 0.05), and the mRNA expression levels of TNF-α, MMP9 and IL-13 were reduced; these results were largely consistent with those of the normal group, with no statistically significant difference.

**Conclusion:**

HKL effectively improved the general symptoms and tissue injury in UC rats, and the therapeutic effect was better than that of 5-ASA group. Ulcerative colitis in rats increased the expression of TNF-α, MMP9 and IL-13. HKL repaired UC-induced colonic injury in rats by decreasing the expression of TNF-α, MMP9 and IL-13.

## Introduction

Ulcerative colitis (UC) is a form of inflammatory bowel disease characterised by damage to the colonic mucosa and submucosa. The main symptoms include weight loss, blood in stools, abdominal pain, diarrhoea, increased defecation and colonic bleeding [1, 2]. In recent years, the prevalence and incidence of UC have increased worldwide [3, 4]. The high incidence of UC imposes a serious economic burden on patients and significantly reduces their quality of life. However, the definite pathogenesis of UC remains unclear.

Defects in immune tolerance caused by the loss of mucosal barrier integrity are considered the primary mechanism [5, 6]. Local inflammation of the colonic mucosa can promote T-cell activation, and activated T cells may attack targets in the colonic mucosa of patients with UC, leading to the development of tissue rupture and further aggravating inflammation, thereby creating a vicious cycle [7]. Studies have shown that Th17 cells are present in all colonic segments [8]; their secreted cytokines interleukin (IL)-6 and IL-17 play an important role in colonic immune responses and inflammatory processes [9] and are closely related to the pathogenesis of UC. The loss of immune tolerance leads to a persistent imbalance in the levels of proinflammatory cytokines, such as tumour necrosis factor-α (TNF-α), IL-6, IL-8 and IL-21, as well as the anti-inflammatory cytokine IL-10 and transforming growth factor-β (TGF-β). Tumour necrosis factor-α, IL-6, IL-8 and IL-10 are regarded as UC biomarkers and therapeutic targets. Among them, TNF-α is secreted by such cells as polymorphonuclear neutrophils, lymphocytes, monocytes and macrophages and is involved in inflammatory, metabolic, apoptotic and thrombogenic processes [10]. Tumour necrosis factor-α is first expressed during the inflammatory response by inducing the apoptosis of colonic epithelial cells, while under the regulation of IL-6, TNF-α can stimulate and induce the generation of thrombin, affect colonic mucosal microcirculation and the repair of colonic mucosal ulcers, promote the release of C-reactive protein by regulating the immune response and aggravate the inflammatory response. It is one of the commonly recognised proinflammatory cytokines mediating the pathogenesis of UC [11, 12].

Matrix metalloproteinase9 (MMP9) plays an important role in the degradation and remodelling of the extracellular matrix (ECM) and is confirmed as the main MMP involved in the development of UC [13]. The ECM is the basic framework for cell attachment and is the most important site of cell metabolism. In a normal human body, the degradation and synthesis of the ECM are dynamically balanced, with the ECM’s turnover and connective tissue reconstruction the main processes of UC pathological injury; MMP9 acts mainly on the important components of the ECM, resulting in the damage of colonic tissue, and the mechanism may be achieved by activating P53 to inhibit cell fine growth and promote cell apoptosis [14].

Most traditional Chinese medicines (TCMs) are derived from plants, exhibit high safety in treating diseases (including UC) and are widely used in clinical treatment in Asian countries [15]. Some TCMs have shown good therapeutic effects in the treatment of UC [16–18]. For 30 years, 5-aminosalicylic acid (5-ASA) has been the backbone of therapeutic management in patients with UC. European and American guidelines recommend 5-ASA as first-line therapy for both the induction and maintenance of remission in mild to moderately active UC [19, 20]. Astragaloside inhibits the Th17 cell response in UC mice by remodelling the homeostasis of Th17/Treg cells in UC mice and promoting the Treg cell response. In addition, astragaloside also inhibits the activation of the Notch signalling pathway in colitic mice and prevents and ameliorates oxidative stress injury [21]. Tanshinone alleviates UC by inhibiting the activation of signal transducer and activator of transcription 3 and the differentiation of Th17 cells [22].

Studies have shown that nutgall has anti-inflammatory, anti-decay, sterilising and haemostatic effects [23]. In our previous study, to further improve the therapeutic effect of drugs on UC, our group modified nutgall and added other TCM materials with effects such as oxidation and anti-oxidation to prepare huankuile (HKL). However, the therapeutic effect and mechanism of HKL on UC are still unclear.

In this study, we observed the therapeutic effect of HKL on UC in UC model rats induced by 2,4,6-trinitrobenzene sulfonic acid (TNBS), assessed the effect of HKL and its effect on the levels of inflammatory cytokines TNF-α, IL-13 and MMP9 and preliminarily explored the mechanism of HKL in the treatment of UC.

## Materials and methods

### Animals

Fifty 8 week-old SPF Wistar male rats weighing (220 ± 20) g were purchased from the Laboratory Animal Centre of Xinjiang Medical University (license number: SCXK [Xin] 2016–0003). All experimental animals were managed and used in such a way as to minimise the number of animals used and alleviate pain during the experiment. Ethical approval was obtained from the Animal Ethics Committee of the First Affiliated Hospital of Xinjiang Medical University (2019, No. IACUC-20190226–31).

### Establishment and grouping of the UC rat model

Fifty 8-week-old SPF Wistar male rats were divided randomly into a normal group (*n* = 12) and a UC group (*n* = 38). The UC rat model was established using a TNBS/ethanol enema with reference to [1]. After fasting for 24 h, the rats were anaesthetised by an intraperitoneal injection of 10% chloral hydrate (0.3 mL/100 g). Next, 5% TNBS (Sigma, USA) was dissolved in an equal volume of 50% absolute ethanol, and after paraffin lubrication, it was injected slowly into the colon (about 8 cm from the anus) of the rats with silicone gel catheter with an inner diameter of 1.5 mm. After the injection was complete, the silicone tube was gently removed, and the rats were held in a handstand position for about 1 min to ensure that the TNBS was fully absorbed. Then, the rats were placed into a cage. The normal group was injected with the corresponding volume of enema, which was administered using 0.9% sodium chloride solution. At the end of the modelling process, the rats were placed in clean cages, where they were allowed to wake up and drink freely. On the second day after the model’s establishment, two rats were selected randomly from the UC group, and the successful construction of the UC model was comprehensively confirmed by the general condition of and histopathological changes in the rats’ colons. The rats in the UC group were divided randomly into a negative control group (*n* = 12), an HKL group (*n* = 12) and a 5-ASA group (*n* = 12, positive control group) according to the random number table method.

### Drug preparation and intervention

Huankuile: Eight types of medicinal materials, including nutgall, *Coptis chinensis*, pomegranate flower, amber and tabasheer, were weighed, pulverised and mixed according to different ratios for future use. *Plantago asiatica* and motherwort fruits were soaked in rose dew to prepare the drug extract, and a powdered mixture of medicinal materials was dissolved in the prepared rose dew filtrate at 0.18 g/mL. The single administration dose for the rats was 1 mL/100 g. The drug concentration was determined according to a previous preliminary experiment: high concentration = 1.8 g/kg, medium concentration = 0.9 g/kg and low concentration = 0.45 g/kg. According to the degree of colonic inflammation, lesion depth, crypt destruction and lesion extent, the therapeutic effect of a high concentration was better than that of medium and low concentrations, and it was also shown that a high dose had advantages in the recovery of rats in terms of the number of surviving animals. Therefore, a high concentration of 1.8 g/kg was selected as the drug therapeutic concentration in this study.

### Tissue specimens

The rats in each group were examined histologically 14 days after administration before being euthanised after anaesthesia with ether. The colonic tissue was cut along the mesentery 5 to 8 cm from the anus, and the intestinal contents were flushed with saline. The colonic mucosa was spread face up, and the extent of mucosal damage was observed with the naked eye. Subsequently, the ulcer site of the colonic mucosa was incised, and a part of the cut ulcer site (about 0.5 × 0.5 cm) was placed immediately in 40 g/L of formaldehyde solution for 24 h. The next day, the sections were embedded with paraffin and stained with hematoxylin–eosin. Pathological observations were performed on the sections along with blind reading. The treatment of the cut colon specimens was consistent with the above method.

### Immunohistochemistry

Tissue samples were fixed in 10% formalin, embedded in paraffin, sectioned, deparaffinised in xylene and treated with 3% hydrogen peroxide for 10 min. Then, they were microwave immersed in citric acid for 20 min for antigen retrieval, incubated in normal goat serum for 1 h after cooling at room temperature to prevent non-specific binding and then incubated with TNF-α antibody (1:100; ab1793, Abcam, USA) or MMP9 antibody (1:100; ab76003, Abcam, USA) overnight at 4℃. Staining was examined using a horseradish peroxidase EnVision system (DAB Kit, MXB Biotechnologies). The staining results were blind evaluated by two experienced pathologists using a high-power light microscope (200 ×). The staining intensities and positive cell ratios were scored by a pathologist. Staining intensity was scored from 0 to 3 as 0 (negative), 1 (weak), 2 (moderate) or 3 (strong). The positive cell scores were 0 =  < 5%, 1 = 5–25%, 2 = 26–50%, 3 = 51–75% and 4 = 76–100%. The final staining score was obtained by multiplying the intensity score by the percentage score. The final score was divided into four groups: ( −) staining = final score of 0, ( +) staining = final score of 1–4, (+ +) staining = final score of 5–8, (+ + +) staining = final score of 9–12. For Pearson's Chi-squared analysis, the final score was classified as negative (final score = 0) or positive (final score = 1–12).

### Real-time PCR

Rat colon tissues were fully ground by a homogeniser, and tissue RNA was extracted using a TRIzol kit (Invitrogen, USA). After adding 1 mL of TRIzol, total RNA was extracted from the colonic tissue using the tri-isopropyl alcohol method. The RNA sample concentrations were determined at 260/280 nm using a NanoDrop™ Ultra-micro-spectrophotometer (Thermo, USA), and after identification by a Bio-Rad gel imager (Bio-Rad, USA), 1 μg of RNA was reverse transcribed into cDNA using a Revert Aid First Strand cDNA synthesis kit (Thermo, USA). The rat mRNA sequences of TNF-α (forward: CCACCACGCTCTTCTGTCTA, reverse: GGGCTTGTCACTGGAGTTTTG), IL-13 (forward: CTCGCTTGCCTTGGTGGTCTTG, reverse: TCTGGTCTTGTGTGATGTTGCTCAG) and MMP9 (forward: TCTGCCTGCACCACTAAAGG, reverse: TCGGCTGAGTAGGACAGAA) were queried in the GenBank database. Primers were designed using the IDT primer design website (https://sg.idtdna.com/pages) and synthesised by Sangon Biotech Co., Ltd (Shanghai, China). Finally, 2 μL of cDNA was taken for PCR amplification on an ABI 7500 (Quant Studio TM Real-Time PCR System) system. Pre-denaturation was performed at 95 °C for 30 s, followed by denaturation at 95 °C for 5 s, annealing for 30 s, extension at 72 °C for 34 s, and maintenance at 4 °C for 40 cycles.

### Statistical analysis

We used SPSS 21.0 statistical software, and the data were expressed as the mean ± standard deviation. When the variance was equal, a one-way analysis of variance was used for comparisons between multiple groups, and the least-significant difference method was used for comparisons between two groups. The Kruskal–Wallis H test was used for multiple group comparisons of pathological score grades, and the Mann–Whitney U test for two independent samples was employed for comparisons between two groups.

## Results

### UC increased the expression of TNF-α and MMP9 in the colonic mucosal epithelia of rats

**Fig. 1:**
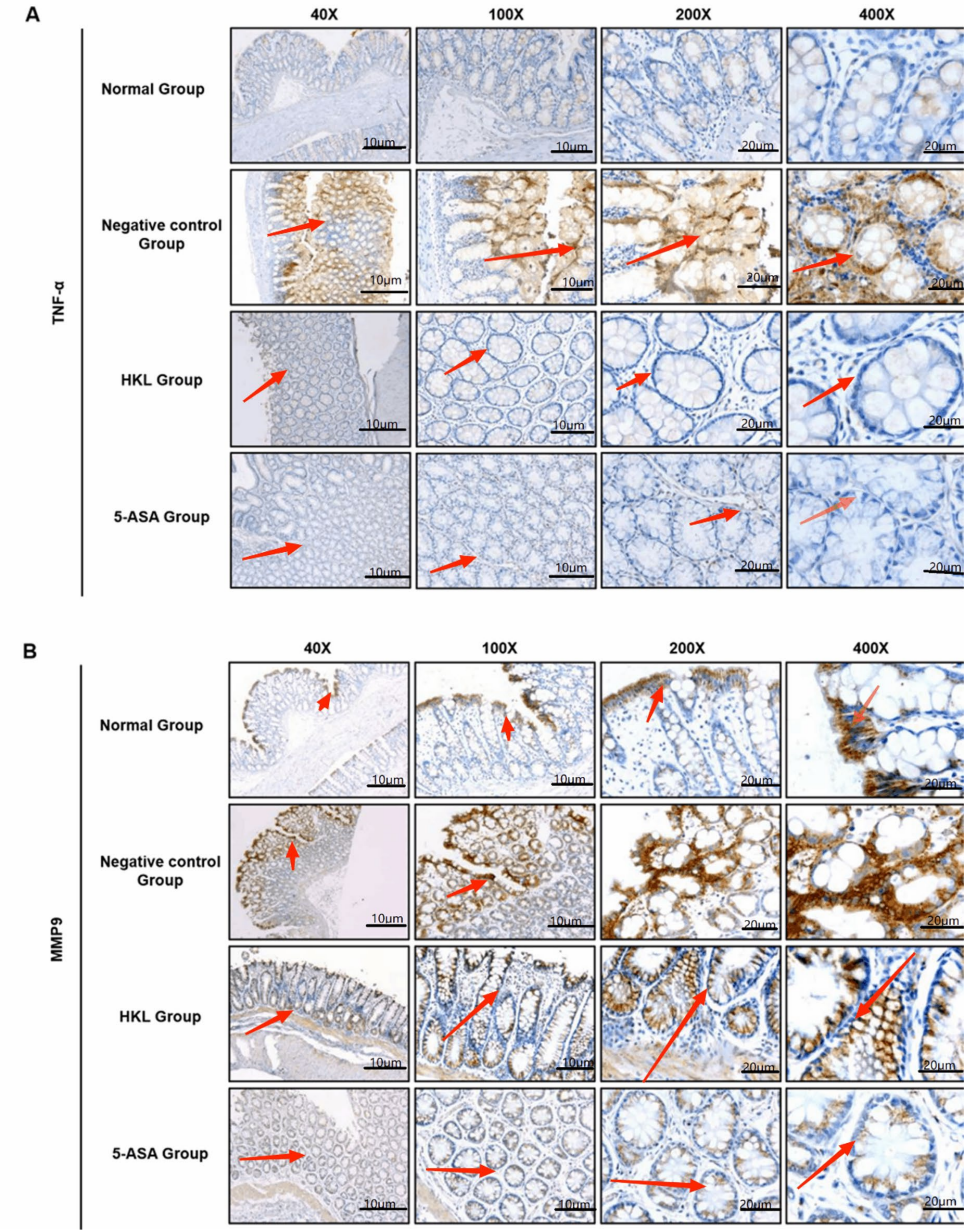
A. Immunohistochemical staining of TNF-α in rat colon under different treatment conditions. B. Immunohistochemical staining of MMP9 in rat colon under different treatment conditions

To further explore the changes in UC protein markers in the colons of rats, our immunohistochemical results revealed that the expression levels of TNF-α and MMP9 in the colonic tissues of rats in the negative control group were significantly upregulated compared with those in the normal group (*P* < 0.05) (Table 1), and the expression locations were concentrated in the colonic mucosal epithelia of the rats (Fig. 1A, B). The above results indicated that UC led to a significant increase in the expression of TNF-α and MMP9 in the colonic mucosal epithelia of rats.

**Table 1:** TNF-α and MMP9 immunohistochemical score analysis

Group	TNF-α	MMP9
Normal group	0.286 ± 0.488	2.571 ± 0.787
Negative control group	3.833 ± 1.722^△^	4.500 ± 0.548^△^
5-ASA group	0.500 ± 0.548^▲^	2.833 ± 0.753^▲^
HKL group	0.222 ± 0.441^▲^	3.111 ± 0.782^▲^

^△^ Means compared with normal group, *P* < 0.05; ^▲^ means compared with negative control group, *P* < 0.05

### HKL decreased the expression of TNF-α and MMP9 in the colonic mucosal epithelia of rats

After treatment with HKL and 5-ASA, compared with the levels in the negative control group, the expression levels of TNF-α and MMP9 in the colonic mucosal epithelia of rats were significantly downregulated (Fig. 1A, B), and the differences were statistically significant (*P* < 0.05). The immunohistochemical scores in the HKL group were lower than those in the 5-ASA group and were largely consistent with those in the normal group (Tables 2, 3). The above results indicated that HKL significantly reduced the expression levels of TNF-α and MMP9 in the colonic mucosal epithelia of UC model rats, and the therapeutic effect of HKL was stronger than that of 5-ASA.

**Table 2:** Differences in distribution of TNF-α immunohistochemistry scores

Group	*n*	TNF-α immunohistochemical score			
		–	+	+ +	+ + +
Normal group	7	5(71.4%)	2(28.6%)	0(0.000)	0(0.000)
Negative control group	6	0(0.000)	2(33.3%)	1(16.7%)	3(50%)
5-ASA group	6	3(50.0%)	3(50.0%)	0(0.000)	0(0.000)
HKL group	9	7(77.8%)	2(22.2%)	0(0.000)	0(0.000)

**Table 3:** Differences in distribution of MMP9 immunohistochemistry scores

Group	*n*	MMP9 immunohistochemical score			
		–	+	+ +	+ + +
Normal group	7	0(0.000)	4(57.1%)	3(42.9%)	0(0.000)
Negative control group	6	0(0.000)	0(0.000)	3(50.0%)	3(50.0%)
5-ASA group	6	0(0.000)	2(33.3%)	4(66.7%)	0(0.000)
HKL group	9	0(0.000)	2(22.2%)	7(77.8%)	0(0.000)

### HKL reduced the mRNA expression levels of TNF-α, MMP9 and IL-13 in the colonic tissues of rats

**Fig. 2:**
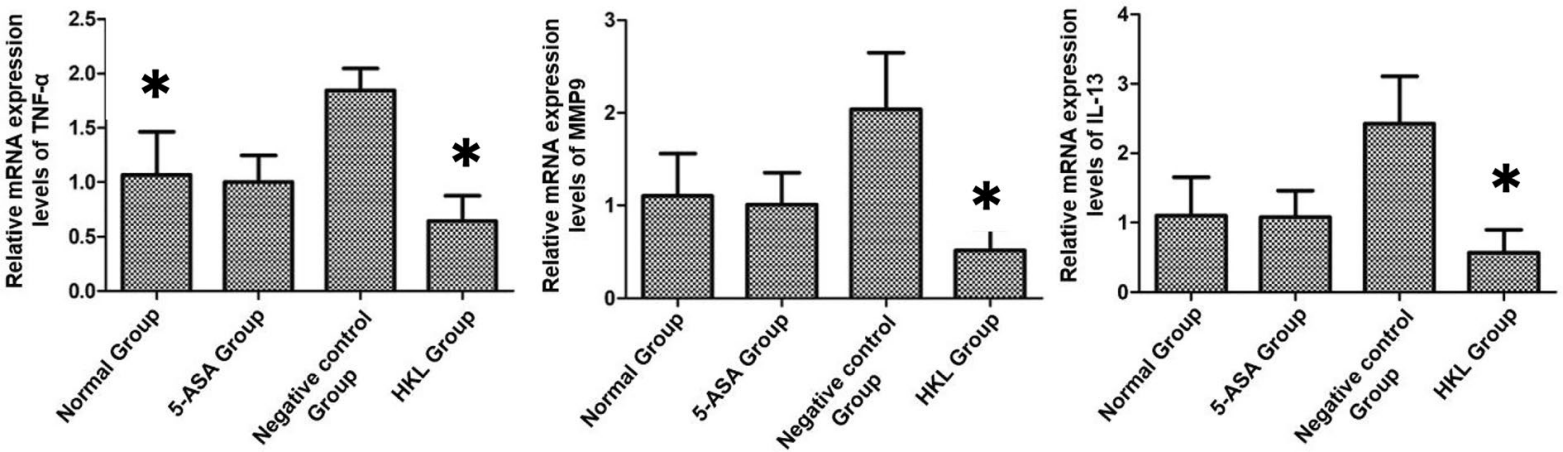
mRNA expression levels of TNF-α, MMP9 and IL-13 in rat colon tissues under different treatment conditions. Data are mean ± SD, *P < 0.05. (TNF-α, tumour necrosis factor-α; MMP9, matrix metalloproteinase 9; IL-13, interleukin-13)

To further explore the molecular mechanism of HKL in the treatment of UC, we investigated the expression levels of related inflammatory factors at the mRNA level (Fig. 2). Compared with the level in the normal group, the mRNA expression levels of TNF-α, MMP9 and IL-13 in the colonic tissues of the rats in the negative control group were significantly upregulated (*P* < 0.05) (Table 4). Compared with the levels in the negative control group, the mRNA expression levels of TNF-α, MMP9 and IL-13 in the HKL and 5-ASA groups were significantly downregulated. The mRNA expression levels of TNF-α, MMP9 and IL-13 in the HKL group were also significantly downregulated compared with those in the normal group (*P* < 0.05) (Table 4). The mRNA expression levels of TNF-α, MMP9 and IL-13 were largely the same in the 5-ASA group and the normal group (*P* > 0.05) (Table 4). The above results showed that HLK significantly reduced the mRNA expression levels of TNF-α, MMP9 and IL-13, and the effect was stronger than that of 5-ASA.

**Table 4:** Analysis of mRNA expression levels in rat colon tissues

Group	MMP9	TNF-α	IL-13
Normal group	1.104 ± 0.456	1.067 ± 0.396	1.100 ± 0.556
5-ASA group	1.008 ± 0.345^▽^	1.003 ± 0.241^▽^	1.080 ± 0.383^▽^
Negative control group	2.038 ± 0.611^△▲^	1.846 ± 0.201^△▲^	2.429 ± 0.679^△▲^
HKL group	0.520 ± 0.231^▽^	0.641 ± 0.234^▽^	0.571 ± 0.324^▽^

^△^ Means compared with normal group, *P* < 0.05; ^▲^ means compared with 5-ASA control group, *P* < 0.05; ▽ means compared with negative control group, *P* < 0.05

## Discussion

In this study, we demonstrated that HKL reduced inflammation in the colon by downregulating proinflammatory cytokines. This lays a foundation for further exploring the potential chemical constituents and therapeutic targets of herbal medicines as well as the promotion and application of HKL in clinical practice.

Ulcerative colitis is an inflammatory disease characterised by lifelong chronic relapse. The degree of progression and the severity of UC are mainly affected by two aspects: first, like most other chronic diseases, various other diseases not only cause UC [24] but also often aggravate the condition and delay the course of the disease, and about half of patients with UC will develop more severe diseases, such as colon cancer. Second, the lack of effective drug treatment often leads to the continued development of UC [25–27]. Therefore, investigating natural drugs for the treatment of UC has become a global research hot spot.

Studies have shown that when colonic lymphoid tissue is stimulated, intrinsic lymphocytes will differentiate into a variety of T cells and cytokines, and some proinflammatory factors, such as IL-1, IL-6, IL-13 and TNF-α, transmit inflammatory signals to inflammatory cells, thereby inducing inflammation. However, the clear pathogenesis of UC is not well understood, and more studies are needed to explore the relevant molecular mechanisms.

Studies have shown that UC leads to metabolic disorders of a variety of inflammatory factors in the colon. Tumour necrosis factor-α is a proinflammatory cytokine that is first expressed in the inflammatory response by inducing the apoptosis of colonic epithelial cells, while under the regulation of IL-6, it can stimulate and induce the generation of thrombin, affect colonic mucosal microcirculation and the repair of colonic mucosal ulcers, promote the release of C-reactive protein by regulating the immune response and aggravate the inflammatory response; it is one of the commonly recognised proinflammatory cytokines mediating the pathogenesis of UC [11, 12]. The above reports are consistent with the results of the present study, and the expression levels of TNF-α and mRNA in colonic tissues decreased significantly after UC was relieved.

Metalloproteinase9 plays an important role in the degradation and remodelling of the ECM and is the main MMP confirmed to be involved in the development of UC [13]. Garg et al. [28] found that MMPs may weaken mucosal defences by regulating goblet cell activity, which causes colonic tissue damage. Santana et al. [29] showed that MMP9 promoted inflammatory cell penetration into inflammatory tissues, thereby aggravating the inflammatory response in the colon of a mouse UC model. Several studies have shown that MMP9 expression levels are significantly increased in colonic tissue and are positively correlated with UC activity [30]. A randomised clinical trial found that patients with UC treated with anti-MMP9 monoclonal antibody (GS-5745) had decreased MMP9 levels in colonic tissue and significantly improved histopathological scores compared with those treated with placebos; this phase-I clinical trial provides both a preliminary basis for the use of GS-5745 in the treatment of UC [31] and a new therapeutic target for UC.

The above studies were consistent with the results of the present study. After treatment with HKL, the expression level of MMP9 in the rats’ colonic mucosal epithelia was significantly downregulated compared with that in the negative control group, and the therapeutic effect of HKL was stronger than that of 5-ASA.

For decades, 5-ASA has been used as the gold standard for the treatment of UC and is still regarded as the first-line drug for the acute treatment and maintenance remission of UC. 5-ASA preparations mainly include sulfasalazine and the new preparations mesalazine, olsalazine (5-ASA dimer) and balsalazide. The main active ingredient exerting the effect of these drugs is 5-ASA. However, 5-ASA is a double-edged sword because of its prominent adverse effects due to excessive oxidative stress, including blood diseases, hepatotoxicity, potential ulcer formation, drug-induced connective tissue diseases, myelosuppression, haemolytic anaemia, megaloblastic anaemia and reversible male infertility [2, 32].

In recent years, TCM has been widely recognised in the treatment of patients with UC based on its multi-target and multi-faceted mechanism of action. There are also many studies reporting the molecular mechanism of a variety of Chinese herbs in the treatment of UC. For instance, Xu et al. reported that colonic injury and submucosal fibrosis were significantly attenuated by treatment with black plum. Reducing the expression of serum inflammatory cytokines TNF-α, IL-8, MMP9 and CXCR-1 protected against TNBS-induced UC in rats and regulated neuropeptides through both oxidative and antioxidant mechanisms [33]. Wang et al. reported that grape seed proanthocyanidin extract increased colonic protection in mice with UC by decreasing the expression of IL-1β, IL-6, TNF-α, nitric oxide and malondialdehyde as well as that of nuclear factor kappa-B and Keap-1 proteins in serum and colonic tissue, thereby playing a role in the treatment of UC [34]. Han et al. reported that HKL significantly reduced proinflammatory cytokines, including TNF-α, IL-1β and IL-6, and inhibited inflammation, thus playing a role in the treatment of UC [35]. This conclusion is largely consistent with the results of the present study. The effect of HKL in UC may be caused by the downregulated expression of endostatin and angiostatin by the modulation of MMP9 via TNF-α inhibition. The inhibition of antiangiogenic factors may represent a novel molecular mechanism of the therapeutic action of 5-ASA.

## Conclusion

In summary, we investigated the therapeutic effect of HKL on UC and its associated molecular mechanisms in a TNBS-induced UC model. The results revealed that HKL significantly reduced proinflammatory cytokine expression. The results of the comprehensive analysis of saline-treated UC and HKL-treated UC samples indicated that immune pathways may be therapeutic targets for HKL. These results shed light on the clinical application of HKL. Further clinical application studies are needed to help demonstrate the pharmacology of HKL.

## Data Availability

All data generated or analysed during this study are included in this published article.
